# Prognostic and Predictive Molecular Biomarkers for Colorectal Cancer: Updates and Challenges

**DOI:** 10.3390/cancers12020319

**Published:** 2020-01-30

**Authors:** Eric Koncina, Serge Haan, Stefan Rauh, Elisabeth Letellier

**Affiliations:** 1 Molecular Disease Mechanisms Group, Department of Life Sciences and Medicine, Faculty of Science, Technology and Medicine, University of Luxembourg, L-4367 Esch-sur-Alzette, Luxembourg; eric.koncina@ext.uni.lu (E.K.); Serge.haan@uni.lu (S.H.); 2 Centre Hospitalier Emile Mayrisch, Department of Oncology, L-4240 Esch-sur-Alzette, Luxembourg; stefan.rauh@chem.lu

**Keywords:** colorectal cancer, biomarkers, molecular markers, early stage CRC, gene signature, clinical transition, clinical test, challenges

## Abstract

Colorectal cancer (CRC) is a leading cause of death among cancer patients. This heterogeneous disease is characterized by alterations in multiple molecular pathways throughout its development. Mutations in *RAS*, along with the mismatch repair gene deficiency, are currently routinely tested in clinics. Such biomarkers provide information for patient risk stratification and for the choice of the best treatment options. Nevertheless, reliable and powerful prognostic markers that can identify “high-risk” CRC patients, who might benefit from adjuvant chemotherapy, in early stages, are currently missing. To bridge this gap, genomic information has increasingly gained interest as a potential method for determining the risk of recurrence. However, due to several limitations of gene-based signatures, these have not yet been clinically implemented. In this review, we describe the different molecular markers in clinical use for CRC, highlight new markers that might become indispensable over the next years, discuss recently developed gene expression-based tests and highlight the challenges in biomarker research.

## 1. Introduction

The global burden of colorectal cancer (CRC) is anticipated to increase by 60%, with 2.2 million new cases and 1.1 million deaths by 2030 [1]. This mortality can be largely attributed to the dissemination of the disease to secondary organs, with the liver being the most common site of secondary metastasis [2,3]. Therefore, early intervention and successful resection of the primary tumor is vital in order to improve outcomes in CRC patients.

Surgical removal is the treatment of choice for early and locally advanced CRC. Current treatment protocols also recommend the systematic use of adjuvant therapy in CRC patients with lymph node involvement (stage III) (https://www.nccn.org; esmo.org), while adjuvant treatment in stage II disease is limited to clinically high-risk patients and still matter of debate. Since the MOSAIC study (Multicenter International Study of Oxaliplatin/Fluorouracil/Leucovorin in the Adjuvant Treatment of Colon Cancer), oxaliplatin-based adjuvant chemotherapy has been the standard treatment for stage III CRC patients, and combinations of fluoropyrimidines with oxaliplatin led to improved overall survival and reduced risk of relapse in these patients [4,5]. Fifty percent of stage III patients are cured by surgery, whereas 20% of patients will survive due to the addition of adjuvant chemotherapy and 30% will relapse within 2–3 years. Altogether, only 20% of stage III patients benefit from chemotherapy, exposing 80% of patients to unnecessary toxicity [6]. In advanced disease, patients are treated with multimodality approaches including surgery and systemic treatments with chemotherapy and biologicals such as cetuximab and bevacizumab.

Currently, CRC patient prognosis is based on clinicopathological features and largely focuses on the cancer stage at the time of diagnosis. The overall five year survival rate is approximately 90% for stage I; it declines to 70% for stage II, 58% for stage III, and less than 15% for stage IV [7]. Over the last couple of years, further diagnostic factors, such as perineural invasion and poor histological differentiation, bowel obstruction or perforation and advanced tumor stage (T4) at the time of surgery, became widely accepted criteria to identify high-risk patients among early stage CRCs.

Major technological developments in the field of molecular biology have led to the clinical implementation of DNA testing on tumor tissue samples. The search for defined oncogenes in colorectal tumors is a routine. The presence, or absence, of certain genes and mutations and the availability of targeted treatments have improved treatment selection and outcome, although this is so far limited to metastatic disease.

Prognostic/predictive gene panels are commercially available, but are not in routine use in Europe, due to their limited predictive value and lack of clear treatment guidance, as well as their high price. As such, the selection of the most beneficial treatment regimens for CRC patients, especially during early stages, remains a challenge due to the lack of adequate markers. Prognostic markers provide treatment-independent prognostic information on patient outcomes, such as overall survival (OS) and relapse-free survival (RFS). Predictive biomarkers, on the other hand, help guide therapy decisions via treatment response information in biomarker-positive patients compared to biomarker-negative patients. In this review, we will describe the different prognostic and predictive molecular biomarkers currently used, as well as highlighting new potential biomarkers that might play a future role in the surveillance and treatment of CRC patients. Finally, we will describe the major challenges encountered in biomarker research and its translation into the clinical setting.

## 2. Current Clinical Used Biomarkers in CRC

Most CRC tumors are sporadic (70%–80%), with approximately 20% being of hereditary origin [8]. CRC is considered a heterogeneous disease and is known to be derived from the accumulation of genetic and epigenetic alterations [9]. The genes most commonly mutated in CRC patients are *APC* (about 80%–82% of cases), *TP53* (48%–59%), *KRAS* (40%–50%), and *PIK3CA* (14%–18%) [10]. Recently, new guidelines for the testing of molecular biomarkers in CRC tumor tissues were established, in order to assist in disease prognosis, surveillance, and treatment. Some biomarkers are based on the mutational status of genes known to be important in CRC carcinogenesis (*NRAS*, *KRAS*, *BRAF*) or associated with defects in the DNA mismatch repair system (MMR) (Table 1). These defects are the underlying mechanism through which microsatellite instability (MSI) status is determined [11].

### 2.1. Microsatellite Instability (MSI) Status

Microsatellites are short tandem repeats of DNA sequences located throughout the genome. MSI status results from a deficient DNA mismatch repair (MMR) system, commonly caused by the inactivation of the four MMR genes *(MSH2, MLH1, MSH6* and *PMS2*). A deficient MMR system leads to a failure in the correction of the insertion or deletion of repeating units during DNA replication, leading to a hypermutable phenotype (MSI-high is characterized by instability at two or more loci). MSI status can be determined by two distinct methods—immunohistochemistry analysis (IHC) or PCR. [31]. Reduced expression of the *MLH1, MSH2, MSH6*, and *PMS2* genes, determined by immunohistochemistry analysis, identifies tumors as MSI (microsatellite instable, also referred to as deficient MMR, dMMR) in contrast to MSS (microsatellite stable, also referred to as proficient MMR, pMMR). Alternatively, standard PCR can be used to compare microsatellite length in tumors versus normal tissue in order to determine aberrant microsatellite lengths detected in the tumor.

MSI tumors can be observed in approximately 15% of all CRC patients [12]. Of the 15%, 3% are associated with Lynch syndrome, an inherited cancer syndrome associated with a genetic predisposition to CRC, also known as hereditary non-polyposis CRC (HNPCC). MSI was initially implemented as a screening method for the detection of the Lynch syndrome [32]. The other 12% of MSI tumors are due to sporadic hypermethylation of the promoter of the MLH1 gene. Of note, the prevalence of MSI is stage-dependent. In stage II/III CRC, up to 15% are dMMR, whereas only 4%–5% of stage IV CRCs are dMMR [33].

MSI tumors are distinct in terms of clinical and pathological features; they are more frequent in the right colon, are more often associated with a younger age and show poor differentiation with a strong lymphocyte infiltrate. Overall, MSI-high patients show a better prognosis compared to MSI-low (MSS) patients [11,31,32]. Recently, the addition of the DNA mismatch repair status to clinicopathological variables has improved prognostic predictions in several cancer types and specifically in CRC patients, leading to its inclusion into the NCCN and ESMO guidelines [44]. It has been suggested that MSI stage II patients do not require chemotherapy, as they seem to have a better prognosis and no beneficial effect of 5-FU has been observed in this subgroup [34,35,36,45]. However, MSI status was only retained as a valuable prognostic marker in localized CRC as its predictive value is not clearly established yet [34]. A meta-analysis composed of 5998 patients from 19 different studies has cast some doubts on the usage of MSI status as a determining factor for the postoperative management of stage II CRC patients, as they found no significant link between MSI status and overall or relapse-free survival [37]. However, a very recent large meta-analysis, including 38 studies with 12,110 patients, further establishes the prognostic significance of MSI status in stage II CRC [46] and indicates the need to implement MSI screening for all resected stage II CRC patients. The MSI status is less informative in stage III patients, as the risk differences are limited between MSI-high and MSS patients [35,38]. Interestingly, patients with MSI tumors and large deletions in *HSP110 T17* show an improved response to 5-FU-based chemotherapy [47].

With the onset of a new era of onco-immunology and the success of checkpoint inhibitors in different tumor types, such as melanoma and non-small-cell lung cancer, MSI status in CRC patients has gradually become a factor of significant interest for a number of researchers and clinicians. Emerging data suggest that tumors with MMR defects respond better to checkpoint inhibitors [48], likely due to their higher mutational load and immune cell infiltration [49]. In 2017 the US Food and Drug Administration (FDA) approved pembrolizumab, a monoclonal anti-PD1 antibody, for use in MSI-high patients, independent of cancer type [50]. Additionally, Nivolumab and Ipilimumab are approved for refractory stage IV MSI-high patients [51]. MSI status is the first biomarker-only based indication for therapy, independent of the primary cancer. Importantly, MSI status might become a predictive marker for stage III MSI-high patients. Indeed, given the significant benefit of checkpoint inhibitors in MSI-high metastatic patients, new trials have started to test immunotherapy, as a stand-alone or in combination with chemotherapy, in stage III MSI-high CRC (ATOMIC trial, NCT02912559).

Nevertheless, not all mCRC patients respond to immunotherapy within the MSI-high patients and, given the high costs of these drugs, further predictive biomarkers are urgently needed to identify intrinsic and acquired resistance. PD-L1 expression in tumors did not predict better survival outcomes in patients treated with immunotherapy, which questions the use of PD-L1 as a predictive marker for checkpoint-inhibition therapy in mCRC [52]. Studies to identify biomarkers in this growing area of clinical research are needed [51].

### 2.2. Mutational Status of RAS

*KRAS* is a downstream effector of the epidermal growth factor receptor (EGFR). In CRC patients, *KRAS* mutations are present in 45% of metastatic tumors [53] and approximately 15%–37% of early stage tumors and is more often found in pMMR compared to MSI tumors. Through epidemiological cohort studies, *KRAS* mutations were suspected to predict outcome in CRC patients [54]. This prognostic value in stage III pMMR [13,55,56], but not MSI [13], tumors was confirmed by post hoc analysis of data collected from adjuvant clinical studies, including studies of trials PETACC-8 and N0147. Initially, only *KRAS* codon 12 mutations (in particular, c.35G > T, also known as G12V), but not codon 13 mutations, were associated with inferior survival in *BRAF* wild-type CRC [56,57,58]. More recent data now support the poor prognosis of both exon mutations [13,59]. In these latter studies, a 1.5 higher risk of relapse and death was found in *KRAS* mutant patients compared to KRAS wild-type patients. Assessing *RAS* mutational status in non-metastatic CRC might contribute to understanding the lack of efficacy of anti-EGFR therapies in early-stage CRC. In addition, mutations in *KRAS* and *BRAF* (for *BRAF* cf paragraph below) are associated with inferior progression-free survival (PFS) and overall survival (OS) of metastatic CRC (mCRC) patients compared to patients with non-mutated tumors. Five randomized trials were used to evaluate the prognostic value of *KRAS* mutations and a total of 1239 CRC patients with metastases from five randomized trials (FIRE-1, FIRE-3, AIOKRK0207, AIOKRK0604, RO91) were included in the analysis [53]. In this meta-analysis, more frequent *KRAS* exon 2 variants, i.e., G12V and G12D did not have a significant impact on OS, whereas the *KRAS* G12C-variant was associated with a lower OS when compared to the non-mutated tumors (multivariate HR 2.26 (1.25–4.1), *p* = 0.001). A similar trend for OS was observed in the KRAS G13D-variant (multivariate HR 1.46 (0.96–2.22), *p* = 0.10).

At present, most advanced patients are treated with multi-modality approaches, including surgery and systemic treatments (reviewed in [60]). The addition of an anti-EGFR biological (cetuximab and panitumumab) to the standard chemotherapy regimen has been shown to improve survival, as well as reduce the risk of disease progression, when compared to singular chemotherapy treatment [3,39,61]. However, this benefit is limited to patients who do not present mutations in downstream effectors of EGFR, such as *KRAS* and *NRAS*, due to the constitutive activation of the downstream MAPK pathway [40,62,63,64]. As activating mutations in *KRAS* and *NRAS* occur in approximately 40% and 7% of CRC patients, respectively [14], mutational analysis is mandatory prior to treatment with anti-EGFR antibodies. The mutational analysis should include *KRAS* and *NRAS* codons 12 and 13 of exon two, 59 and 61 of exon three, and 117 and 146 of exon four (https://www.nccn.org).

Importantly, not all wild-type *KRAS* patients respond to anti-EGFR treatment, and the potential emergence of therapy resistance is an important issue. Anti-EGFR therapy leads to the development of *KRAS*, *NRAS*, *BRAF* and *EGFR* ectodomain mutations, which drive the MAP kinase pathway activation despite EGFR inhibition. Studies in which patients were re-challenged with anti-EGFR agents showed an improved overall response rate, mostly likely due to the fact that resistant clones decay exponentially after drug removal [65]. Additional biomarkers are needed in order to select the patients amongst the wild-type *KRAS* population who are likely to respond to anti-EGFR therapy, as well as to identify those that have become resistant, as this type of personalized treatment is often extremely expensive.

### 2.3. Mutational Status of BRAF

The BRAF gene is activated by mutations in 10% of CRC [66]. BRAF-activating mutations most frequently occur in codon 600 (BRAF V600E), which represents almost 90% of all BRAF mutations [67,68]. This mutation is typically mutually exclusive with other RAS mutations [40,69]. BRAF p.V600 mutational analysis is recommended in patients with pMMR-positive tumors that display a loss of MLH1. In these patients, the *BRAF* p.v600 mutation excludes Lynch syndrome [32].

Kalady and colleagues combined 21 studies including 9885 CRC patients. They concluded that *BRAF*-p.V600-mutated tumors are often associated with four or more positive lymph nodes, high-grade histology, MSI status, higher prevalence in females, and are often located in the right side of the colon, while wild-type tumors can be found in any part of the colon [70]. Several retrospective studies underlined that microsatellite stable (MSS) patients with *BRAF* mutations have more than a two times greater risk of relapse and mortality than those with wild-type *BRAF* [14,15,16,17,18,19]. In recent studies, the presence of *BRAF* mutations was found to reduce patient survival in stage III and IV (objective response rate (ORRs) <10%, with a PFS of about two months, and OS of four to six months [20,21]) but not stage II CRC [41]. Although larger studies are needed, these recent results do not support the testing of *BRAF* status in stage I and II CRC. Additionally, there is currently no evidence that patients with *BRAF*-mutated tumors are less likely to benefit from standard chemotherapy agents (irinotecan and oxaliplatin in the MRC FOCUS Trial) [18]. Altogether, testing the mutational status of *BRAF* p.V600 has until very recently (BEACON study, see below) been exclusively a prognostic marker for stage III-IV CRC, with little impact on therapy decision.

Interestingly, in most of the early *BRAF* studies, MSI status was not clearly included in the analyses. It is now clear that MSI *BRAF*-CRC and MSS *BRAF*-CRC show different prognoses and outcomes, with a shorter OS and RFS in *BRAF*-MSS patients while no difference is observed in MSI *BRAF*-CRC compared to wild-type patients [12,13]. These results clearly suggest that the *BRAF*-CRC subtype should not be defined as one entity [68]. Along this line, the response of *BRAF* mutant tumors to targeted anti-BRAF strategies remains limited and varies extensively within *BRAF* V600E cohorts. This heterogeneity in drug resistance might be explained by biologically distinct subpopulations within *BRAF*-mutated tumors. Accordingly, Barras and colleagues recently identified two subtypes based on gene expression data, BM1 and BM2, which are independent of MSI status, PI3K mutation, gender and sidedness. Whereas BM1 subtype is characterized by KRAS/AKT pathway activation, mTOR/4EBP deregulation and EMT, BM2 shows important deregulation of the cell cycle. Further dissection of the heterogeneous motifs of *BRAF*-mutated tumors might be exploited for biomarker development, as well as for drug targeting [71]. In the future, the identification of further subgroups of *BRAF*-CRC might help clinicians choose more beneficial therapies, as standard treatment regimens are not sufficient for *BRAF*-MSS patients.

Several studies have suggested that *BRAF* mutations (which are *RAS* wild-type and may therefore benefit from anti-EGFR therapy) predict the lack of response to anti-EGFR treatments in CRC [22,39,40,42]. Two further meta-analyses validated this finding in *KRAS*-wild type mCRC patients [23,24]: the addition of anti-EGFR antibodies in *BRAF* mutant mCRC patients did not lead to an improved outcome compared to the standard therapy or best supportive care. This notion was recently challenged by a meta-analysis performed by Rowland and colleagues. The authors concluded that there is currently insufficient evidence to definitively state that *KRAS* wild-type/BRAF-mutated metastatic tumors respond differently to anti-EGFR therapy compared with *KRAS* wild-type/*BRAF* wild-type tumors [43]. Thus, data concerning the response to EGFR-targeting agents in *BRAF*-mutant CRCs remain conflicting.

As mentioned previously, *BRAF* inhibition strategies in metastatic *BRAF*-mutated individuals have shown a dismal outcome. Indeed, pre-clinical studies have suggested that *BRAF* inhibition in CRC leads to the robust adaptive feedback of signaling networks, including the activation of EGFR, leading to the reactivation of MAPK signaling and sustaining tumor growth [25,26]. As a follow-up, pre-clinical studies combining anti-EGFR and/or MEK or HER therapy with BRAF inhibitors were performed and demonstrated promising results [27,28,29], which led to the initiation of clinical trials [25,26]. Recently, the phase-three-study BEACON CRC showed that patients with *BRAF* V600E mutated mCRC benefit from the doublet or triplet chemotherapy-free targeted combination therapy of encorafenib (a BRAF inhibitor), and cetuximab (an anti-EGFR antibody) or the latter ones combined with binimetinib (a MEK inhibitor) in a second or third line setting [30] (http://clinicaltrials.gov/show/NCT02928224). This has led to FDA approval of both the doublet and triplet therapy as a treatment for patients with advanced *BRAF*-V600E-mutated mCRC following up to two prior lines of therapy. This approval is a breakthrough, with a chemotherapy-free targeting combination in a difficult subpopulation of CRC patients. The magnitude of clinical benefit may, however, be overrated, due to a debatable control arm of the BEACON study, which has been the subject of criticism. Further trials are underway to validate this new combinatorial strategy in the first line setting.

Importantly, given the overlap of *BRAF* V600E mutations with a high number of MSI, checkpoint inhibitors may play an important role in this CRC cohort. For all of the above-mentioned reasons, and to further shed light on the value of *BRAF* as a biomarker, NCCN guidelines have recommended routine testing of *BRAF* mutational status in advanced mCRC patients.

## 3. Future Promising Biomarkers

In this chapter, we will present biomarkers, which show promising results and might therefore translate into the clinical setting (please refer to Table 2).

**Table 2:** List of promising future biomarkers in CRC.

Biomarkers	Value	Therapy Involved	Target Group	Ref.
**Currently Translated into Daily Routine**				
ctDNA	Predictive/prognostic	√ Targeted therapies	All CRC	[72,73,74,75,76,77,78,79]
		√ Chemotherapy duration		
Tumor sidedness	Prognostic/predictive	× Anti-EGFR therapy (RCC)	All CRC/mCRC	[80,81,82,83]
		√ Intensive Chemotherapy (RCC)		
		√ Immunotherapy (RCC) (most likely)		
ALK, ROS1, NTRK1-3 fusions	Predictive	√ ALK, ROS and TRK inhibitors	All CRC ALK, ROS and NTRK fusions	[84,85,86,87,88]
HER2 amplification	Predictive	√ Anti-HER2 strategies	All CRC with HER2 amplification	[89,90]
		× Anti-EGFR strategies		
**Need for Further Investigation**				
CMS subtyping	Prognostic	Predictive value currently studied	All CRC	[12,91,92,93,94]
Immunoscore	Prognostic	Predictive value not yet determined	All CRC	[95,96,97,98,99,100]
Markers based on the stromal compartment	Prognostic/predictive	× Adjuvant chemotherapy in rectal cancer	All stages with a focus on stage II for the prognostic value	[101,102,103,104,105]
Tumor mutation burden	Predictive	√ Immunotherapy	mCRC	[77,78,79]
CIMP	Prognostic	Predictive value not yet determined	All CRC	[106,107,108]
PI3K	Predictive	× Anti-EGFR therapy	mCRC	[40,109,110,111]
		× First-line chemotherapy	All CRC	
miR-31-3p (low expression)	Predictive	√ Anti-EGFR therapy	KRAS wt mCRC	[112,113,114,115,116]
**Promising Biomarker Specifically Focusing on Stage II CRC**				
CDX2 (low expression)	Prognostic	Predictive value not yet confirmed	Stage II CRC	[117]
MYO5B (low expression)	Prognostic	Predictive value currently studied	Stage II CRC	[118]

Benefit: √; No benefit: ×.

### 3.1. Liquid Biopsy: ctDNA and Tumor Mutation Burden

Promising tools, such as liquid biopsy, have the ability to provide clinically relevant information (reviewed in [72]). Liquid biopsy refers most of all to the collection and analysis of circulating tumor cells, cell-free nucleic acids and tumor-derived exosomal vesicles, which are released by the primary or metastatic site of the tumor into the bloodstream or other fluids. The identification of ctDNA can be challenging: whereas ctDNA can form up to 50% of total cell-free DNA in later metastatic stages, it can represent less than 1% or even be undetectable in earlier tumor stages. The potential for liquid biopsy in guiding treatment and monitoring the disease is compelling and may soon be translated to clinical practice. In recent years, ctDNA has been shown to be a powerful tool in (1) assessing the adequacy of surgical tumor clearance and thereby the risk of recurrence (2) in selecting the most appropriate targeted therapy and (3) in following responses to systemic treatments [73,74]. The reappearance or increase in ctDNA, along with the emergence of new mutations, is associated with recurrence, progression and resistance to therapy. Therefore, ctDNA measurement emerges as a more sensitive method for monitoring disease progression compared to current clinical tools. Once the mutational profile of a given patient has been identified by tumor biopsy, this unique profile can be used to follow disease progression with ctDNA measurements in a personalized way.

Several studies have suggested the possibility of using ctDNA to closely monitor patients after surgery and identify patients with a high risk of recurrence. The independent prognostic value of ctDNA was presented in a recent phase III trial (IDEA-France). In this trial, patients with positive ctDNA four weeks after primary surgery were associated with a poor outcome compared to negative ctDNA patients when given a three months adjuvant therapy [75]. In addition to different mutations, methylated markers such as SEPT9 may be used as surrogate markers for the detection of residual tumor burden following surgical resection [76].

The tumor mutation burden, which can be assessed using ctDNA, was recently suggested to identify responders to checkpoint blockade therapy [77,78]. It is not yet clearly established whether TMB is an independent prognostic factor. Additionally, the definition of TMB, as well as the method used to assess it, can vary extensively between laboratories. Recently, it has been suggested that adding MSI and ctDNA to TMB might increase the prediction efficiency of checkpoint inhibitors [79]. Further upcoming studies involving larger cohorts are needed to validate these findings.

### 3.2. Tumor Sidedness

One of the most intriguing concepts in mCRC is the impact of the primary tumor location (PTL). It has been known for decades that the colon has two distinct embryological origins, namely the midgut for the proximal colon (also referred to as right-sided colon) and the hindgut endoderm for the distal colon (also referred to as left-sided colon). Additionally, the two parts of the colon have different blood supplies, distinct microbiome populations and are associated with different biological features [80]. Although the dogma is not fully accepted yet within the scientific community, several studies support the concept that right-sided colon cancer (RCC) shows a worse prognosis than left-sided colon cancer (LCC) [81,82]. Importantly, post-hoc analysis of the CRYSTAL and FIRE-3 trials, suggests an association between PTL and response to anti-EGFR therapy [83], as right-sided *KRAS* wild-type CRC did not seem to benefit from cetuximab treatment. Additional studies need to analyze PTL in order to definitively accept it as an independent predictive biomarker for anti-EGFR therapy, especially as RCC and LCC are also characterized by different mutational landscapes. Nevertheless, based on the results mentioned above and according to current guidelines, anti-EGFR therapy should be limited to left-sided *KRAS* wild-type CRC. LCC seems to benefit more from adjuvant chemotherapies such as 5-FU based regimes [80], whereas RCC might justify more intensive chemotherapy treatments in the metastatic setting. Additionally, RCC suggests more promising results with immunotherapies, as these tumors display a high antigenic load [80].

### 3.3. NTRK, ALK and ROS

Overall the incidence of gene fusion in CRC is in the range of 0.5%–2% in CRC patients; however, their prognostic and predictive value is far from being elucidated [84]. Promising results from the STARTRK study revealed that entrectinib, a small molecule which selectively inhibits ALK, ROS1 and TrkA-B-C, was able to induce impressive results in heavily pre-treated mCRC patients harboring LMNA-NTRK1 [85], CAD-ALK [86] and STRN-ALK fusions [87]. As ALK fusions have recently been shown to be involved in resistance to BRAF inhibitors in melanoma [88], combinatorial therapies combining ALK inhibitors with other targeted therapies might lead to some therapeutic benefit in a subset of CRC patients. Similar to the approval of pembrolizumab in MSI tumors, the FDA approved the NTRK inhibitor entrectinib in NTRK-fusion-mutated tumors of all organ types, including CRC, provided they do not harbor a known acquired resistance mutation, in 2019.

### 3.4. HER2 Aberrations

HER2 (Errb2) is a transmembrane receptor of the EGFR family and its activation leads to cell proliferation and apoptosis inhibition. *HER2* overexpression is due to *ERBB2* amplification or the activation of somatic mutations and is defined in clinical practice as IHC 3+ or IHC 2+ and ISH-positive disease. Among CRC patients, the frequency of *HER2* overexpression is reported to be around 5%, with ERBB2 amplifications reported in 5.5% [89]. Recently, HER2 gained a lot of interest in CRC, as two recent clinical trials, MyPathway (trastuzumab and pertuzumab) and HERACLES (trastuzumab and lapatinib), demonstrated promising clinical benefit for dual HER2 blockade in patients with HER2-amplified mCRC (reviewed in [89,90]). The prognostic value of HER2, which is more frequently observed in sigmoid tumors, is still under debate and has recently been reviewed in Auclin et al. [6]. There is currently more interest in HER2 as a predictive marker for anti-EGFR therapy. Data from several studies suggest that acquired amplifications of ERBB2 negatively predict efficiency and are associated with the development of resistance to EGFR-targeted therapies (reviewed in [89,90]). The implementation of HER2 assessment in daily practice might provide useful information for guiding therapy decisions.

### 3.5. Consensus Molecular Subtypes

Genomic information has recently gained prominence as a potential alternative to clinicopathological features for determining the patient’s risk of relapse. In contrast to breast cancer, the identification of various genetic subgroups of CRCs have so far been disappointing when applied as prognostic markers. In late 2016, a large consortium of several groups working on CRC combined their efforts and identified four molecular subtypes based on multi gene arrays and conserved across all examined studies [12]. These subtypes are referred to as CMS1 (MSI-immune subgroup representing 14% of CRC cases), CMS2 (canonical subgroup accounting for 37% of cases), CMS3 (metabolic subgroup representing 13% of CRC patients) and CMS4 (mesenchymal representing 23% of CRC cases) [12]. CMS subtyping primarily shows an association with clinical outcomes [91]. Besides CMS classification, Isella and colleagues have proposed five CRC intrinsic subtypes (CRIS) which are defined by unique molecular, functional and phenotypic features [92]. Even if some recent pre-clinical studies have highlighted the clinical relevance of CMS and CRIS subtypes by demonstrating differing drug efficacy between tested subtypes [93,94], the clinical impact of the definition of these subtypes remains relatively limited.

### 3.6. Markers of the Tumor Microenvironment (TME)

#### 3.6.1. Immune Cell Infiltration

It is becoming increasingly evident that the tumor microenvironment has an important role in disease progression and tumor resistance. Along this line, the infiltration of tumors by lymphocytes has been suggested as a prognostic marker [95,96,97]. Based on this observation, Galon and collaborators introduced the Immunoscore classifier, which assesses the presence of CD3+ and CD8+ lymphocytes within the tumor and invasive margin [98,99]. Indeed, patients with tumors in which these lymphocytes can be detected (which are also called “hot” tumors) exhibit better relapse-free survival times than patients with tumors devoid of these immune cells (“cold” tumors). Non-infiltrated tumors can be further subdivided based on the presence of lymphocytes in the invasive margin (suggesting that immune cells might be attracted by the tumor but unable to enter), placing the patient in an intermediate risk status. Hence, tumors are classified as low, intermediate and high Immunoscore (with low Immunoscore being non-infiltrated and placing patients at risk). A recent international study, performed on a cohort of 3539 patients, validated the scoring system [99]. Interestingly, while most MSI tumors are infiltrated [96,100], the Immunoscore has been reported to be a better predictor than MSI alone [100]. However, associating the Immunoscore to all currently available clinical parameters showed only a modest, albeit significant, increase in predictivity [99]. Additionally, the efficacy of the Immunoscore in predicting response to immunotherapy agents has not yet been demonstrated [100].

#### 3.6.2. Stromal Density

The tumor-stroma percentage has been confirmed as a prognostic factor in stage II and III CRC (VICTOR trial). OS and DFS were significantly lower in patients with a high percentage of tumor stroma [101]. In addition to the quantity of tumor stroma, its composition may be an important determinant of cancer behavior. For example, the presence of cancer-associated fibroblasts (CAFs) effectively predicts tumor recurrence in CRC patients [102]. Recently, an immune-histochemical score based on the expression of two proteins specific for CAFs has additionally been able to predict the response to neoadjuvant treatment in rectal cancer [103]. Nevertheless, CAFs have been shown to be highly heterogeneous and better markers are currently needed to identify subtypes and shed light on their potential prognostic/predictive value in CRC [104]. The combination of stroma quantification with a functional activity assessment of CAFs might lead to an improved stroma-based tool for patient stratification [105].

### 3.7. CpG Island Methylator Phenotype (CIMP)

CpG islands are genomic regions that contain a high number of cytosines and guanine nucleotides, which are located in 5’ regulatory promoter regions. The CpG island methylator phenotype (CIMP) has been recognized as one mechanism of CRC tumorigenesis. The methylation of CpG islands in the promoters of genes involved in malignant transformation leads to the CIMP phenotype, which is present in approximately 18% of CRC patients. The common molecular alterations *KRAS*, *BRAF* and *TP53*, as well as the MSI status, are often associated with CIMP. The hypermethylation of at least three out of five pre-defined markers defines CIMP. There are only a limited number of studies that have assessed the prognostic value of CIMP: two retrospective monocentric studies [106,107] and one post hoc analysis of the CALGB 89803 prospective trial [108]. While these three studies suggest that CIMP+ tumors have worse survival compared to CIMP tumors, additional studies are needed, especially as CIMP status seem to overlap with *BRAF* mutations and MMR status. Thus, the independent prognostic value of CIMP needs to be validated.

### 3.8. PI3KCA

Approximately 14%–18% of CRC patients have mutations in the *PI3KCA* gene [119]. *PIK3CA* mutation hot spots are located at five sites in exons nine and 20 [40], which are oncogenic in CRC models [120]. A recent meta-analysis covering twenty-eight studies enrolling 12,747 patients did not demonstrate a substantial prognostic role of *PIK3CA* mutation status in CRC [109]. However, several reports suggest that *PI3KCA* mutations, especially in exon 20, are linked to clinical resistance of anti-EGFR therapies [40,110] and to first-line chemotherapy [111]. It is difficult to evaluate the importance of PIK3CA as an independent predictive marker [40] as *PIKC3A* mutations often co-occur with *RAS* or *BRAF* mutations. Large cohorts, including patients harboring mutations in *PIKC3A* but not in *RAS* or *BRAF*, are needed in order to shed light on the value of PIKC3A as a biomarker in CRC. Therefore, routine testing for *PIK3CA* is currently not recommended. Importantly, PIK3CA inhibitors currently show promise in the treatment of some hematologic and breast neoplasias, but not in colorectal cancer.

### 3.9. TP53

*TP53* is the most frequent somatic gene mutation in all cancer types. The mutational status of *TP53* has been associated with a positive response to adjuvant 5-fluorouracil therapy in stage III CRC patients [121]. Interestingly, in metastatic CRC, patients with *TP53* mutations receiving adjuvant therapy displayed poorer survival outcomes [122]. More studies are necessary in order to determine the role of TP53 as a potential prognostic and predictive biomarker in CRC.

### 3.10. miRNAs

miRNAs are small, non-coding RNA molecules which play an important role in the regulation of intracellular processes via the post-transcriptional regulation of gene expression [123]. miRNAs are considered to be exceptional biomarkers due to their involvement in multiple physiological pathways and their stability in paraffin-embedded (FFPE) tissues, which is an important factor for the translation of biomarkers into the clinics. Recently, miR-31-3p expression has been proposed as a promising predictive biomarker for anti-EGFR therapy in KRAS wild-type patients treated with adjuvant chemotherapy. Low expression of miR-31-3p in patients treated with chemotherapy and cetuximab has been correlated with longer progression-free survival when compared with patients expressing high levels of miR-31-3p [112,113,114]. After several validation studies from over 850 patients from nine independent patient cohorts [115,116], a qPCR-based test, called miRpredX, has been developed by IntegraGen.

## 4. The Need for Biomarkers in Early-Stage CRC Patients

Surgical resection is the treatment of choice for early CRC stages. In stage II patients, the use of adjuvant chemotherapy remains highly controversial, as surgical resection is typically sufficient to limit recurrence in the vast majority of CRC patients. Therefore, treatment of stage II CRC patients with chemotherapy is often considered to be an “overtreatment” as only a subset of patients will benefit from it. Although adjuvant chemotherapy improved overall survival of stage II CRC patients in the QUASAR study, the absolute improvement in overall survival (OS) was limited (about 3.6% [124]). Nevertheless, up to 30% of stage II patients will relapse after surgery and many of these patients will succumb to their disease [7]. It is therefore important to identify these high-risk patients to provide them with adequate therapies. Additionally, it is also important to identify patients who do not require these treatments and who can be treated with other, less arduous and costly, methods.

In recent years, a large amount of effort has been invested in the identification of high-risk stage II patients who might benefit from adjuvant chemotherapy. Both NCCN (https://www.nccn.org) and ESMO (https://www.esmo.org) guidelines have identified several clinical factors that predict poor patient prognosis, including emergency presentation (tumor obstruction, perforation), the inadequate number of assessed lymph nodes (<12), T4 tumors, poor histological differentiation, lymphovascular invasion, perineural invasion, and the presence of positive resection margins. Three prognostic scores, MSKCC, ACCENT and Numeracy, have been elaborated on the basis of these clinical and pathological features [6]. Additionally, as mentioned previously, primary tumor location seems to act as an important prognostic factor, recently reviewed in Gallois et al. [125]. However, these clinicopathological factors are insufficient and the identification of early-stage CRC patients at high risk of relapse is an unmet clinical need.

### 4.1. Tests Based on Gene Expression

Recently, several biomarkers based on single or multigene expression signatures have been proposed for identifying high-risk subgroups in early-stage CRC patients. A limited number of these molecular markers are currently commercially available for oncologists (Table 3). OncotypeDX, commercialized by Genomic Healthcare, uses a 12-gene assay, which includes seven cancer-related genes and five reference genes, detected by RT-PCR on formalin-fixed, paraffin-embedded tumoral tissues [126]. The second genome classifier, ColoPrint, is an 18-gene expression profile developed by Agendia, a molecular diagnostic company [127]. ColoPrint uses patient RNA profiles obtained from fresh/snap-frozen specimens or samples preserved in special preservative solutions, such as RNA *later*. Both genome classifiers have been proposed to be capable of predicting the development of distant metastasis in stage II CRC patients and to identify patients who may be safely managed without chemotherapy, independently of other clinical risk factors. Other commercial kits, such as OncoDefender [128] and GeneFX [129] (Table 3), are also available, but possess a far lower market share than OncotypeDX. ColoGuideEx [130] and ColoGuidePro [131], first published in 2012, are two prognostic gene expression signatures for stage II and III CRC, respectively. However, no commercial test is currently available for these two classifiers, suggesting that the translation from a microarray-based platform to a PCR-based platform is challenging for larger multi-gene signatures. In addition, several recent studies have highlighted the poor performance of commercially available gene classifiers, the only exception being OncotypeDX [132,133,134]. Recently, a large meta-analysis comprising 2166 samples from 12 independent datasets was set up in order to assess different molecular gene signatures for their capacity to predict patient survival. While ColoGuideEx, ColoGuidePro, Oncodefender, and Coloprint did not show any significant association with survival, OncotypeDX was able to significantly predict survival (*p* = 6.6×10^−2^, HR = 2.05) [133]. However, when comparing the prognostic efficacy of the kit to that of MSI status, gender, KI67, and CDX2 expression, OncotypeDX lost its independent prognostic value. Furthermore, OncotypeDX has a number of major limitations, chiefly the high cost of the assay (€3200) [135] and the complex scoring system [136]. More specifically, OncotypeDX uses three categories: low, intermediate, and high, with only high patients identified as high-risk. This leaves clinicians with a high number of patients for whom treatment decision remains questionable, as an intermediate category can affect up to 39% of the patients [137], leading to a small degree of discrimination between the low- and high-risk groups (22% of recurrence in the high risk group vs. 12% in the low-risk group [136]). In addition, OncotypeDX appears to be a better prognostic marker for stage III disease (which is universally considered for adjuvant treatment) than for stage II patients [134]. In this stage, easily available factors such as T4 and MSI status appear as stronger predictors of recurrence [136,138], making the additional benefit of OncotypeDX less stringent. Therefore, OncotypeDX is unlikely to play a major role in determining treatment in stage II CRC patients [138].

### 4.2. Other Promising Markers for Early-Stage CRC (I and II)

Algorithm-based signatures separate between high- and low-risk patient groups without considering the role of the involved genes. Whereas many biomarkers have been described in fundamental research studies, we would like to highlight here two biomarkers, *CDX2* and *MYO5B*, which have been identified based on a similar approach. They both focus on genes which are often associated with the differentiation of epithelial cells. Loss of function of these genes will lead to a lack of differentiation, and consequently to an immature phenotype of cells, which is one of the hallmarks of cancer.

#### 4.2.1. CDX2 Expression

Recently, Dalerba and colleagues identified *CDX2* as a potential prognostic biomarker in stage II CRC patients [117]. In their approach, they studied genes involved in the differentiation of epithelial cells, which led them to identify *CDX2* as an actionable biomarker. Although the results seem encouraging, only 87 out of 2115 (4.1%) CRC patients were tested *CDX2-*negative and, therefore, were considered as high-risk patients. Due to both the small number of high-risk patients and a rather questionable predictive value (*n* = 48 stage II *CDX2*-negative patients were analyzed for chemotherapy efficiency), *CDX2* cannot yet be considered clinically applicable. In contrast to early-stage CRC patients, *CDX2* status is not affected by chemotherapy in the metastatic setting [141]. Further studies are necessary in order to corroborate *CDX2* as a prognostic and predictive marker in stage II CRC patients.

#### 4.2.2. MYO5B Expression

Our group examined potential markers involved in the differentiation of epithelial cells. As *MYO5B* is involved in the differentiation/polarity of epithelial cells [142], we hypothesized that it might be dysregulated during carcinogenesis. Using a bioinformatics approach [143], we analyzed different CRC datasets that covered over 800 CRC patients. We found that *MYO5B* often displays reduced expression in tumorigenic tissue. We validated these results in our own independent CRC cohort [118] by using laser micro dissected material, as well as tissue microarrays from paired human CRC samples (tumor and normal counterpart of the same patients). Importantly, CRC patients with low *MYO5B* expression displayed shorter overall, disease-, and metastasis-free survival. Altogether, *MYO5B* displayed a very strong prognostic value in stage II patients [118], making it a potentially promising standardized clinical test to help clinicians decide whether to administer adjuvant treatment. Further validation studies are planned to fully elucidate the prognostic and predictive power of this biomarker.

## 5. Challenges in Biomarker Research and Clinical Translation of Biomarkers

The translation of cancer biomarkers into clinical practice is challenging, but of utmost importance in order to attribute the most efficient treatment to well defined patient groups and thus improve patient outcomes. In recent years, a number of studies have described promising correlations between molecular features and treatment results. Nevertheless, only a limited number of these have ever seen clinical use [144]. The most common reasons for this discrepancy are the low number of systematic analyses carried out on existing biomarkers, inadequately sized patient cohorts, the lack of an optimal scoring/threshold system, technical differences between laboratories, insufficient analytical validation on proposed assays, and lastly the low number of well-designed validation studies. As these steps often require resources beyond those of the initial pivotal pre-clinical study, they are often lacking or insufficient. A good molecular prognostic biomarker should, therefore, pass the following criteria.

### 5.1. Significant Prognostic/Predictive Value in Large CRC Datasets

The vast majority of identified biomarkers have been initially discovered in small datasets: while identifying gene-signatures, researchers typically use a training set composed of a limited number of patients. This training set is then confirmed in a “validation set”, still carrying a limited number of involved patients. As an example, Coloprint was initially identified in a training set that included 188 CRC patients, and subsequently validated in a “validation set” composed of 206 patients [127]. Two validation studies followed; one used an independent CRC cohort that covered 135 patients [139], and the second one covered 416 patients and was performed by the researchers who initially discovered the signature, but using 135 previously analysed patients [140], limiting the study’s validation potential. A prospective clinical trial (PARSC, https://clinicaltrials.gov/ct2/show/NCT00903565), which was supposed to end in December 2019, will hopefully elucidate the efficacy of Coloprint as a prognostic marker in stage II CRC patients. In conclusion, rigorous validation studies are essential for biomarker validation.

### 5.2. Homogenous Expression Within and Between Tumor Tissues

Cancer is a heterogeneous disease with both inter- and intra-tumor heterogeneity. As such, an ideal biomarker should have a homogenous expression both within and between tumor tissues in order to perform as a sensitive and specific biomarker in all patients. Thus, a panel composed of various biomarkers might be a good approach to address both inter- and intra-tumor heterogeneity. However, until now, multigene-expression signatures have not yet shown consistent results across different patient cohorts (i.e., ColoPrint, ColoGuideEx [132,133,134,136,138]), and thus have not been approved by the FDA. One major limitation of gene signatures identified via microarrays is the heavy reliance on the technology and platforms used, which often generates divergent gene lists. The disparity, caused by the use of different probes and labels between the various platforms, was addressed as an important issue at the FDA’s Microarray Quality Control workshop [145]. Even when identical RNA samples were run on three different commercial platforms, the resulting gene panels showed an extremely high degree of divergence [146]. Thus, it is extremely difficult to identify a robust gene classifier based on microarray-based gene signatures, as different platforms and sample types are often used for their identification. Next-generation sequencing (NGS) may solve some of these issues. However, NGS is still difficult to translate into daily clinical use due to its technical elaboration, expense, availability, and complex application.

### 5.3. Ease of Clinical Translation

Multi-gene expression signatures are often difficult to apply in clinical practice for a number of reasons, such as the proportion of truly differentially expressed genes, the distribution of such differences, and the variability of measurement. In addition, aspects such as the statistical approach and the challenges associated with implementing a robust scoring system also play a key role in slowing down the clinical application of multi-gene expression signatures (national comprehensive cancer network and [147]).

### 5.4. Analytical Validation

Assay development is a critical component of biomarker qualification [148,149]. Potential biomarkers often fail to translate into clinically useful biomarkers, not due to the validity of the underpinning science, but rather due to the lack of validation studies and issues surrounding assay development [144,150]. During analytical validation, sample-processing delays, storage and processing temperatures, and other elements (such as sample types, paraffin vs. fresh frozen), which undermine biomarker robustness, are thoroughly tested. In addition, various analytical characteristics are examined in order to validate the biomarker. This includes aspects such as precision, linearity, carry-over, trueness, interference, the matrix effect, field of application, analytical range, and stability. Overall, analytical validation should confirm that the tested method meets acceptable standards of performance.

### 5.5. Independence from Clinicopathological Features and Sample Type

Conceptually, in order for a gene-based prognostic analysis to be robust, any relevant gene information which is associated with clinicopathological variables should be extracted, in order to clearly demonstrate that the prediction does not require the external addition of information from clinical variables. The choice of sample type (FFPE vs. fresh-frozen) can also lead to further reproducibility problems of a biomarker across different datasets.

### 5.6. Definition of a Threshold

The interpretation of qPCR results requires a robust and comprehensive threshold that can be used in order to categorize patients into one of two categories (low vs. high-risk). Such a cut-off point must also be easily applicable by practicing pathologists. Furthermore, an optimal threshold should be reliable and reproducible between different clinicians. Performing a receiver operating characteristic (ROC) curve analysis (StepMiner algorithm) represents a systematic way of defining a cut-off value. This analysis can assess the prognostic sensitivity and specificity of biomarkers across a range of cut-off points. The selected threshold would then be used in validation studies to assess the accuracy of the biomarker.

### 5.7. Clinical Validation

The validation of potential biomarkers in independent tissue cohorts is often considered to be the most significant factor responsible for delaying clinical application [144]. Many authors start with the validation of the identified biomarkers using retrospective studies, as it is difficult and time-consuming to set up prospective clinical trials for biomarkers. Nevertheless, a prospective clinical study is vital in order to earn the approval of various regulatory authorities. Therefore, more focused and aim-oriented validation studies could expedite the journey of biomarkers from the bench to the clinic.

## 6. Conclusions

Biomarkers can greatly improve the selection of treatment strategies for CRC patients. However, especially in CRC, most of these markers might inform clinicians of the overall prognosis of the disease, but they fail to guide therapeutic decisions. Indeed, most of these biomarkers, except for the *KRAS* and *BRAF* genes and MSI status, are currently not accurately predicting treatment response. As such, additional studies are urgently needed to identify and validate novel biomarkers in order to improve outcomes for CRC patients. Especially with the importance of immunotherapy in cancer treatments, it is increasingly important to determine new biomarkers predicting the efficacy of immune-based therapies in patients.

With the rapid evolution of molecular testing, as well as our constantly improving understanding of CRC and its molecular progression, we will hopefully enter a new era of tailored treatments in which routinely used biomarkers allow for more precise patient treatment. It is most probably a combination of several different biomarkers assessing the tumor mutational landscape and the clinicopathological characteristics, as well as the features of the TME, that will lead to new successes in the biomarker field. Future developments should focus on integrating all of these aspects.

## Figures and Tables

**Table 1:** Current clinical used molecular biomarkers in colorectal cancer (CRC).

Status	Prevalence	Prognostic Value	Predictive Value		Ref.
			Non-Metastatic	Metastatic	
Microsatellite instability (MSI) high	overall: 15% stage II/III: 15% stage IV: 4.5%	Increased overall survival in stage II	× 5-FU monotherapy (stage II) √ FOLFOX (stage III)	√ Immuno-therapy	[11,12,13,14,15,16,17,18,19,20,21,22,23,24]
*KRAS/NRAS* mutation	45%	Bad prognostic (debatable in stage II)	No impact on treatment	× Anti-EGFR therapy	[25,26,27,28,29,30,31,32,33,34,35,36,37,38]
*BRAF* mutation	7–10%	Bad prognostic especially in the metastatic setting	Currently no impact on treatment	× Anti-EGFR therapy (conflicting data)	[12,13,14,15,16,17,18,19,20,21,22,23,24,25,26,27,28,29,30,39,40,41,42,43]
				√ Combi therapy: Anti-BRAF/Anti-EGFR	

Benefit: √; No benefit: ×.

**Table 3:** List of industrially developed or currently available commercial prognostic gene signatures for CRC.

Name	Company	Genes/Gene Signature	Targeted Population (Stage)	Actual Status	Ref.
ColoPrint	Agendia	*MCTP1, LAMA3, CTSC, PYROXD1, EDEM1, IL2RB, ZNF697, SLC6A11, IL2RA, CYFIP2, PIM3, LIF, PLIN3, HSD3B1, ZBED4, PPARA, THNSL2,* and *CA438802*	II	Not FDA approved	[127,139,140]
OncotypeDX	Genomic Health	*BGN, MKI67, MYBL2, GADD45B, FAP, INHBA,* and *C-MYC*) and five reference genes (*ATP5E, GPX1, PGK1, UBB*, and *VDAC2)*	II/III	Available test Not FDA approved	[124,130,131,132,133,134,135,136]
ColDX/GeneFX	Almac/Helomics Therapeutics	634 probe set	II/III	Available test Not FDA approved	[129]
OncoDefender	Everist Genomics	*BMI1, ETV6, H3F3B, RPS10*, and *VEGFA*	I/II	Available test Not FDA approved	[128]
ColoGuideEx	inven2	*PIGR, CXCL13, MMP3, TUBA1B, SESN1, AZGP1, KLK6, EPHA7, SEMA3A, DSC3, CXCL10, ENPP3, BNIP3*	II	Unknown status	[130]
ColoGuidePro (derived from ColoGuideEx)		*OLFM4, CXCL9, DMBT1, UGT2B17, SEMA3A, NT5E*, and *WNT11*	III	Unknown status	[131]
